# Small membranous proteins of the TorE/NapE family, crutches for cognate respiratory systems in Proteobacteria

**DOI:** 10.1038/s41598-018-31851-2

**Published:** 2018-09-11

**Authors:** Olivier N. Lemaire, Pascale Infossi, Amine Ali Chaouche, Leon Espinosa, Silke Leimkühler, Marie-Thérèse Giudici-Orticoni, Vincent Méjean, Chantal Iobbi-Nivol

**Affiliations:** 1 Aix-Marseille Université, Laboratoire de Bioénergétique et Ingénierie des Protéines, Institut de Microbiologie de la Méditerranée, Centre National de la Recherche Scientifique, 13402 Marseille, France; 2 Aix-Marseille Université, Laboratoire de Chimie Bactérienne, Institut de Microbiologie de la Méditerranée, Centre National de la Recherche Scientifique, 13402 Marseille, France; 3 Institute of Biochemistry and Biology, Department of Molecular Enzymology, University of Potsdam, 14476 Potsdam, Germany

## Abstract

In this report, we investigate small proteins involved in bacterial alternative respiratory systems that improve the enzymatic efficiency through better anchorage and multimerization of membrane components. Using the small protein TorE of the respiratory TMAO reductase system as a model, we discovered that TorE is part of a subfamily of small proteins that are present in proteobacteria in which they play a similar role for bacterial respiratory systems. We reveal by microscopy that, in *Shewanella oneidensis* MR1, alternative respiratory systems are evenly distributed in the membrane contrary to what has been described for *Escherichia coli*. Thus, the better efficiency of the respiratory systems observed in the presence of the small proteins is not due to a specific localization in the membrane, but rather to the formation of membranous complexes formed by TorE homologs with their *c*-type cytochrome partner protein. By an *in vivo* approach combining Clear Native electrophoresis and fluorescent translational fusions, we determined the 4:4 stoichiometry of the complexes. In addition, mild solubilization of the cytochrome indicates that the presence of the small protein reinforces its anchoring to the membrane. Therefore, assembly of the complex induced by this small protein improves the efficiency of the respiratory system.

## Introduction

A few years ago, Storz *et al*.^1^ claimed that “small proteins can no longer be ignored”. Indeed, the importance of small proteins in both prokaryotes and eukaryotes rises according to their number and their involvement in various cellular functions. Small proteins are defined as proteins of around 50 amino acids that are the product of small open reading frames in the genome and are not derived by proteolytic processes^1^. Often it is difficult to decipher the precise role and the accurate mechanism of action of the small proteins. Due to their size, it is accepted that small proteins do not possess an enzymatic activity. However, functions of these small proteins are versatile. For instance, small proteins are involved in the sporulation process like SpoVM of *B*. *subtilis*^2^, affect signal transduction like MgrB and Sda^3,4^, act like chaperones as described for MntS^5^ or FbpB^6^ and regulate inner membrane transporter like MgtS^7^ as well as the activity of membranous enzymatic complexes like CydX^8–12^. They are located in the various compartments of the cell, soluble in the cytosol like MntS in *Escherichia coli*^5^ and Sda in *Bacillus subtilis*^3^, membrane-associated like SpoVM in *B*. *subtilis*^2^ and embedded in the membrane as MgtR^13^ and CydX^11^ in *Salmonella enterica* and *E*. *coli*. CydX optimizes the activity of the cytochrome *bd* oxidase CydAB complex by interacting with it. In *E*. *coli*, deletion of the gene *cydX* leads to a decrease of the respiratory activity of the complex while it is abolished in *Brucella abortus cydX* deletion mutant^8,9^. Furthermore, it is proposed that CydX has a direct role in the positioning and stability of the hemes contained in the CydAB complex but no influence on the complex stability^10,11^. CydX is a hydrophobic, small structured alpha-helical protein integrally located in the membrane, and surprisingly its topology in the membrane differs from one organism to another^8,10,11^.

Recently, a small protein called TorE has been reported in the aquatic bacterium *S*. *oneidensis*^14^. The 56 amino acid protein TorE has been reported to be part of the Tor respiratory system that reduces trimethylamine oxide (TMAO) into the volatile and odorous trimethylamine (TMA)^15^. TMAO, widely spread in marine environments, has an important role in fish to counteract urea and pressure damages and it is considered as a kosmotropic chaperone^16–18^. TMAO is also used as a respiratory substrate by various bacteria and especially *Shewanella* sp.^15,19,20^. TMAO reduction is mainly due to the Tor system, which production is strictly controlled by the presence of TMAO in the bacterium environment^14,15^. It is made up by the periplasmic TMAO reductase TorA and the pentahemic membrane-anchored *c*-type cytochrome TorC^15,21^. The latter is organized in two domains; the tetrahemic domain anchored to the membrane belongs to the NapC/NirT cytochrome family^22^, it interacts with the TMAO reductase TorA, receives the electrons from the quinone pool and transfers them to the second domain which in turn feeds TorA catalytic site^23,24^. In addition to the genes encoding TorA, TorC and TorE, the *tor* operon contains a fourth gene, *torD* that codes for the specific chaperone of TorA^25,26^. It has been shown previously that TorD is required for TorA stability and that it belongs to the family of chaperones specifically dedicated to molybdoenzymes of the DMSO reductase family to which TorA belongs^27,28^. In *S*. *oneidensis*, the absence of TorD leads to the absence of TorA activity and degradation of the protein^14^. In contrast, in the absence of the small protein TorE the activity of the Tor system is significantly decreased, although the maturation, concentration and location of TorA and TorC are not modified^14^. Furthermore, a fitness experiment revealed that TorE presence gives a strong selective advantage to the bacteria harboring TorE^14^.

Here we report that TorE interacts with the *c*-type cytochrome TorC and allows for the formation of a 220 kDa-membrane complex, with a potential 4:4 stoichiometry that gives TorC a better anchor into the membrane stabilizing the Tor system, thereby increasing its catalytic efficiency. Thus, TorE plays indirectly a key role in the electron transfer of the Tor system. Moreover, TorE and its homolog NapE from the periplasmic nitrate reductase respiratory system are part of a family of small proteins that are required to stabilize membrane anchored respiratory systems of certain proteobacteria.

## Results

### Synteny study of TorE family proteins

**Figure 1:**
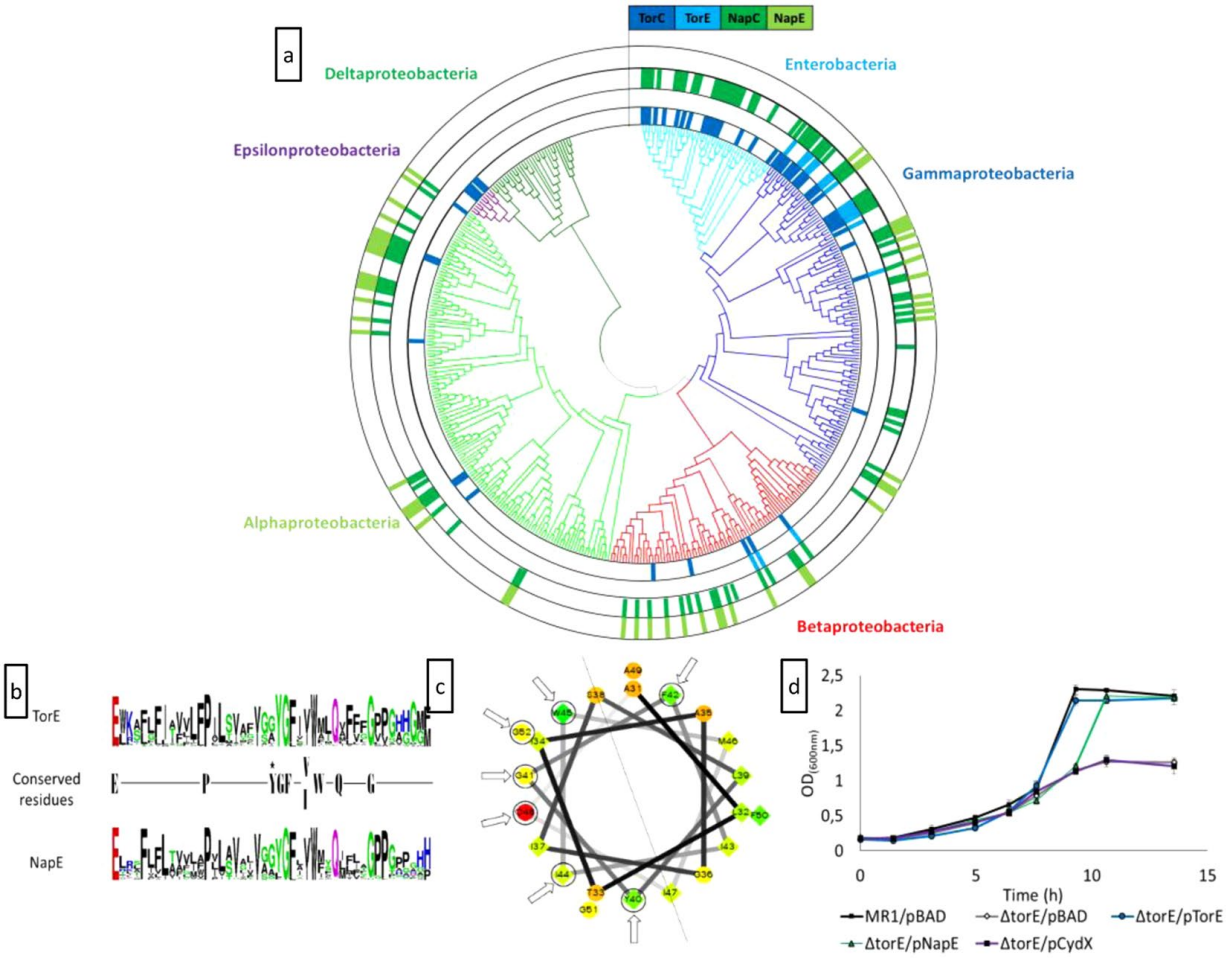
(**a**) Occurrence of torC, torE, napC and napE genes among the proteobacteria. The presence of each gene is represented by block coloured in dark blue, light blue, dark green and light green, respectively. The phylogenetic tree has been constructed using the GyrB protein sequence from a bacterium of each genus of proteobacteria. Branches are coloured according to the class of proteobacteria. Within the gammaproteobacteria class, the enterobacteria order was specifically indicated. (**b**) Sequence logo of the TorE and NapE proteins. The conserved residues in the two sequences are shown. The black star indicated that the tyrosine residue is highly conserved in the NapE proteins except in bacteria that also possess the TorE protein. (**c**) Helical projection of the hydrophobic stretch of the TorE protein of *S. oneidensis*. These residues (from 31 to 52) were selected because of their location between two prolines. The conserved residues are indicated by arrows. The centreline of the helix is represented. (**d)** Complementation experiments. Growth curves of the wild type strain (MR1/pBAD) and ΔtorE mutant harbouring either pBAD, pTorE, pNapE or pCydX plasmid. Cells were grown anaerobically in LB medium in the presence of TMAO, arabinose and chloramphenicol. Each point represents the average ± standard error of three independent experiments.

The small protein TorE contains 56 amino acids. It is a hydrophobic protein with a calculated molecular mass of 6119.29 Da and a pI of 9.52 (Expasy tools^29–31^). As many integral membrane small proteins, TorE is predicted to contain a single hydrophobic α helix spanning through the membrane (TMHMM tool)^32,33^. A bioinformatic analysis using TorE from *S*. *oneidensis* as a probe indicated that TorE homologs are found in synteny with a multihemic *c*-type cytochrome of the NapC/NirT family (Fig. 1a) and a molybdoenzyme. TorE homologs can be divided into two subfamilies: first, the TorE orthologs in synteny with pentahemic *c*-type cytochromes homolog to TorC and second, the TorE homologs, NapE, in synteny with tetrahemic *c*-type cytochrome NapC (Fig. 1a). NapE and NapC are part of the alternative nitrate respiratory system Nap, which terminal reductase NapA allows the reduction of nitrate in the periplasmic space of bacteria. The absence of NapE leads to a decrease of the Nap specific activity^34^. It is noteworthy that TorE orthologs are found in various genders of β and γ classes of Proteobacteria, whereas in α and ε-Proteobacteria the systems are present in a few genders and do not possess any TorE homolog. Compared to the Tor system, the Nap system presents a higher distribution in all division of proteobacteria except the epsilon and delta proteobacteria, with the latter not synthetizing either of these two systems. Except for enterobacteria, the Nap system contains in most cases NapE (NapENapC/NapC ratio of 68%), while TorE is less distributed in the phylogenetic tree (TorETorC/TorC ration of about 43%). Surprisingly, while in Enterobacteria, the Tor and Nap systems are well distributed, with none of them containing the small TorE/NapE proteins. One hypothesis could be that these proteins were lost during the evolution under conditions that do not require a highly efficient respiratory system. The comparison of the consensus sequences of both subfamilies shows that the proteins share homologies only in their hydrophobic stretch and that nine amino acids are highly conserved (Fig. 1b). Moreover, using a simulation program, it seems that in both proteins these residues are located on the same side of the α-helix with the sole exception of the highly conserved residue Phe_42_ (Fig. 1c). This indicates that the sequence variability is high for these proteins as long as the location of the conserved residues is maintained in the structure.

### NapE improves the growth of a *torE* deleted strain

To corroborate these observations, we tested whether it is possible to replace TorE by NapE in the Tor system. Since *S*. *oneidensis* has no NapE and NapC proteins, we used *napE* gene from *S*. *massilia*^15^. Thus, a *torE* mutant was generated that was complemented with either *torE* or *napE*. The recombinant and mutated strains were incubated with TMAO as sole substrate for respiration and their growth was followed and compared to that of the wild type strain. The *torE* mutant shows a growth defect that was complemented with the *torE* gene as previously shown^14^. Interestingly, the *torE* mutation was also complemented by *napE*, although a growth delay was observed. Nevertheless, this complementation indicates that NapE can replace TorE in the functionality of the Tor system. To verify that this result is specific to TorE/NapE family, we tried to complement the *torE* mutation by *cydX* coding for a small transmembranous protein involved in the cytochrome *bd* oxidase CydABX of *S*. *oneidensis*^11^. In the latter case, the growth defect of the recombinant strain is still observable, showing that CydX in contrast to NapE cannot replace TorE in the Tor system. This is also in agreement with the fact that no CydX protein was found among TorE or NapE homologs, since they do not share any conserved residues although CydX is a small membranous protein involved in a respiratory system.

### Membrane localization of TorE

**Figure 2:**
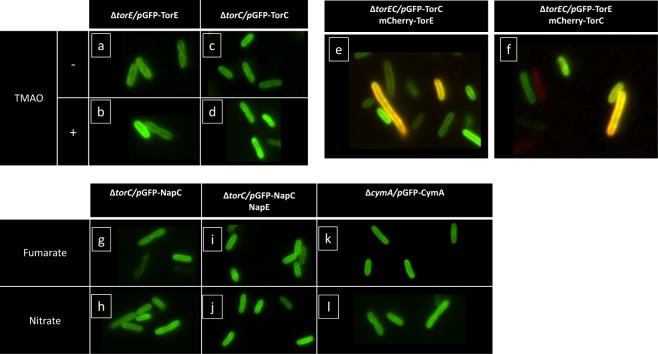
Fluorescence microscopy of *S. oneidensis* MR1 cells producing GFP and mCherry fused respiratory proteins. The genetic context of the strains, the plasmids harboured (pBAD33 derivatives) and the fluorescent chimeras are indicated in each panel. Cells were grown in a rich medium supplemented with fumarate (panels: a, c, e, f, g, i, k) or with electron acceptors inducing specific respiratory systems of *S. oneidensis*: TMAO (panels b and d); nitrate (panels h, j, l) and that are indicated on the edge of the panels. TMAO (−) stands for no TMAO added in the medium preventing chromosomal gene expression and thus the production of the Tor components.

At the moment, many researches are focused on how proteins are organized in the bacterial membrane. In the membrane of prokaryotes, microdomains are structured to increase the efficiency of the response to environmental cues. They are made up of lipid rafts harboring various proteins like flotillin and proteins involved in signaling and transport^35^. In *Pseudomonas aeruginosa*, denitrification occurs through a membrane located supercomplex containing the four multisubunit enzymes allowing the production of molecular nitrogen from nitrate reduction and also many other enzymes and proteins^36^. In *E*. *coli*, the nitrate reductase NarGHI has been described as present in the membrane mostly at the cell poles under anoxia, allowing a higher nitrate reduction rate under certain conditions^37^. Since the presence of TorE increases the efficiency of the respiratory system, we wondered whether TorE could play a role in the targeting to a particular localization of the Tor system in the cell membrane. In a first approach, a functional chimeric protein GFP-TorE was engineered and produced in a strain deleted of *torE* gene (Figure S1). The subcellular localization of the GFP-labeled TorE was characterized by fluorescence microscopy imaging on exponentially growing *S*. *oneidensis* cells under fumarate-respiring conditions (Fig. 2a). The fluorescence signal was only detected at the edge of the bacteria and it was evenly distributed. By fractionation of the cells, we confirmed that the fluorescence was entirely in the membrane fraction and not in the periplasm. To test whether the presence of the other components of the Tor system may influence the localization of TorE, cells were grown in the presence of TMAO, the latter allowing the production of the TorA and TorC proteins from the chromosomal gene expression. The fluorescence signal was similarly distributed and no specific cluster of TorE was obvious (Fig. 2b). To corroborate these results, the same approach was performed with functional TorC labelled with GFP and produced in a strain deleted of *torC* (Figure S1). The fluorescence signal was well distributed along the membrane of cells grown in the presence or absence of TMAO, indicating that the cytochrome has no particular localization in the membrane whatever the presence of TorE and of TorA which production is strictly induced by TMAO^14,15^ (Fig. 2c,d). Finally, when TorE and TorC linked to the GFP and the mCherry tags were produced concomitantly from a plasmid without *torA* induction, their distribution along the membrane was still unchanged indicating that the absence of the terminal reductase TorA has no effect on the distribution of either TorC or TorE. Altogether these results indicate that the Tor respiratory system has no special spatiolocalization in the membrane as seen with the NarGHI complex^37^. Moreover, the presence of TorE does not induce a clustering of the Tor system in the cell membrane. To extend these conclusions, a similar approach was performed with NapE and NapC of *S*. *massilia* produced in *S*. *oneidensis*. The experiments were done with GFP labelled-NapC produced in the presence or absence of NapE. Fluorescence microscopy imaging indicated that NapC was uniformly distributed along the membrane and that the concomitant production of NapE has no effect on it. Finally, the distribution of CymA, another tetrahemic *c*-type cytochrome of the NapC/NirT family, was tested. CymA has no known small protein associated and is involved in several respiratory chains in *S*. *oneidensis* acting as the quinol oxidase during the reduction of fumarate, DMSO, nitrate and other electron acceptors, except TMAO^38–41^. The presence and localization of CymA was tested in cells grown with various substrates showing that it was evenly distributed whatever the nature of the electron acceptor (Fig. 2h).

### TorE reinforces TorC anchoring to the membrane

**Figure 3:**
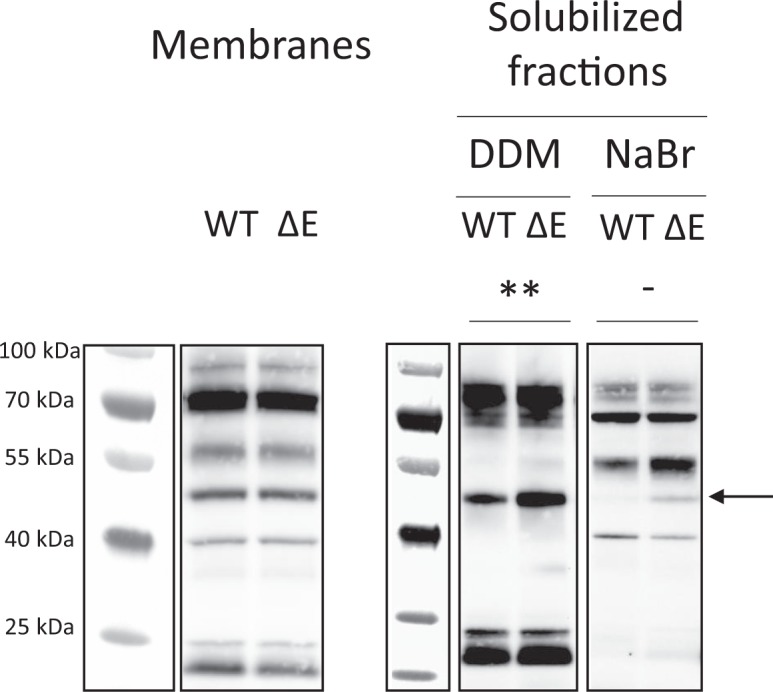
Solubilization of the TorC cytochrome under mild conditions. Peroxidase activity of *c* type hemes of cytochromes from membranous and membrane extracted proteins of *S. oneidensis* wild type cells (WT) or ΔtorE deletion mutant (ΔE) either after 2% DDM solubilization or 1.5 M NaBr. Ten microgram proteins were loaded in each lane of the 3–14% SDS gel. After electrophoresis, c-type cytochromes were revealed by their peroxidase activity. TorC is indicated by an arrow. **T test analysis performed by GraphPad Prism Software shows that data from three replicates are significantly different (p = 0.0058). No band can be detected in the WT lanes for three replicated experiments, preventing statistical analyses Separated portions of the same blots are represented with their respective molecular weight markers (see Figure S2).

We have shown in a previous work that TorE increases the efficiency of the Tor system and gives a selective advantage to the strain when competing with a *torE* mutant^14^. This suggests that the electron transfer is more efficient in the presence of TorE which can be due to a direct effect of TorE on either TorC prosthetic groups i.e. one of the five hemes, on the quinol binding site of TorC or on the stabilization of TorC and the whole respiratory system. To determine whether TorE acts also on the spectral properties of TorC, we expressed either *torC* or *torEC* in *E*. *coli* grown anaerobically with fumarate as sole source of electron acceptor. Under these conditions, the unique *c*-type cytochrome produced would be TorC of *S*. *oneidensis*^42^. Since the redox spectrum of TorC was similar for membranes where TorE was present or absent, this suggested that TorE has no effect on TorC spectrum. The stabilization of the Tor system by TorE was also investigated. We looked for conditions under which TorC anchoring into the membrane was reinforced by the presence of TorE. To do so, the membranes were treated either with a non-ionic detergent (n-Dodecyl-β-D-maltoside) or with a salt (NaBr) and the supernatants obtained after the solubilization were loaded on SDS-gel to decipher the amount of TorC released in the presence or absence of TorE (Fig. 3). We observed that when the membranes were incubated with DDM for a short time (20 min), the amount of TorC recovered in the solubilized fractions is higher when TorE is absent than in the wild type strain. Moreover, when membranes were treated with NaBr, allowing the release of proteins weakly anchored and the solubilized fraction submitted to SDS-PAGE, a band corresponding to TorC is observed only in the sample in which TorE was absent. This result suggests that the release of TorC by salt extraction or after a rapid solubilization is facilitated in the absence of TorE and thus, that TorE improves the anchoring of the cytochrome into the membrane.

### TorE and TorC constitute a membranous complex

**Figure 4:**
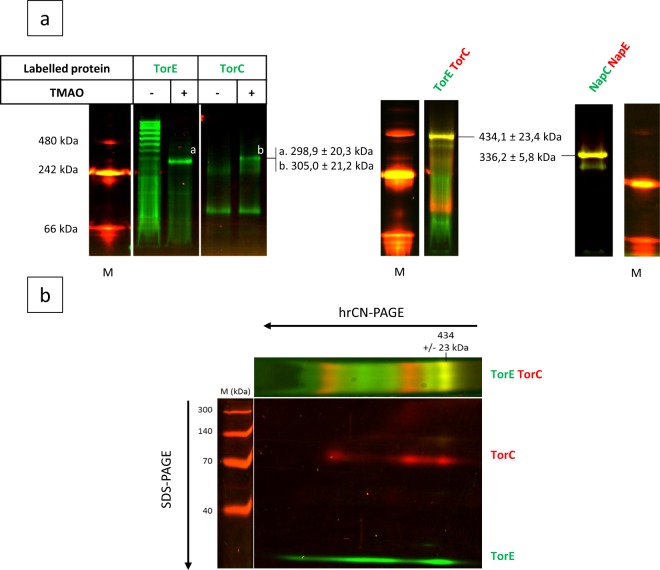
*In vivo* identification of the TorE-TorC complex. (**a**) hrCN-gels, first dimension. The strains were growth in anaerobiosis. The fluorescent labelled proteins were solubilized from the membrane extracts of the various recombinant strains using DDM (2%, v/v). The solubilized fractions were loaded on hrCN gel (3–14%) and in-gel detection of fluorescent labelled proteins and complexes was performed by scanning. Recombinant strains Δ*torE*/pGFP-TorE (TorE) and Δ*torC*/pGFP-TorC (TorC) were grown in rich medium supplemented with TMAO to induce the other components of the Tor system (+). The strain Δ*torEC* carrying either pGFP-TorE-mCherry-TorC (TorE-TorC) or pmCherry-NapE;GFP-NapC (NapE-NapC) were grown in rich medium. Separated gels are represented with their respective molecular weight markers. White lines separate portions of the same initial gel (see Figure S3). (**b**) SDS-Gel, second dimension. A band of hrCN gel corresponding TorE-TorC was excised and after treatment with SDS and β-mercaptoethanol was submitted to SDS-12%-PAGE. The direction of the migration is indicated by arrows. (**a**,**b**) Fluorescence was revealed by scanning. Complexes TorE-TorC and NapE-NapC are indicated by arrows. M is for molecular weight markers. The pictures are representative of three independent experiments.

Taking into account the solubilization results and the fact that TorE spans the membrane, our hypothesis is that TorE could stabilize TorC and better anchor it to the membrane by interacting with it. We decided to visualize the interaction of the two proteins directly on membranous extracts. In a strain deleted of *torE* gene, we produced GFP-labelled TorE in the presence or not of TMAO and the solubilized membrane fraction was loaded on a 3–14% high resolution Clear Native (hrCN) gel. After electrophoresis, the GFP-labelled TorE was revealed by appropriate laser scanning. When the membrane extract was from the cells grown without TMAO, the fluorescence obtained indicated that TorE presents numerous states of oligomerization, while when the extract was from cells grown in the presence of TMAO, only one fluorescent band with a molecular weight of about 300 kDa was observed (Fig. 4). Since the addition of TMAO during the cell growth induces the expression of the remaining *tor* genes, i.e. *torCAD*, and since only TorC is anchored to the membrane, our hypothesis was that GFP-TorE interacts with TorC to create a complex. To verify this, the reverse experiment was performed. In this experiment, the *torC* deleted strain harboring a plasmid allowing the production of GFP-TorC was grown in the presence or absence of TMAO corresponding to the presence or absence of TorE in the extract, respectively. When GFP-TorC was produced without the other Tor proteins, two bands were observed: a major band at 70 kDa corresponding to the chimeric GFP-TorC and a faint band that could correspond to a trimer of GFP-TorC (Fig. 4a). When TorE is produced in the extract a third band appears on the track corresponding to a complex with a molecular weight of ≈305 kDa. To corroborate these results, we produced concomitantly both proteins labelled with GFP and mCherry for TorE and TorC, respectively. In addition to the bands corresponding to the mCherry-TorC and the GFP-TorE, a band with a combined fluorescence was revealed that corresponded to the complex of the two chimeras with a mass of about 434 kDa (Fig. 4a). To gain insight into the protein profile of the complex, a two-dimensional hrCN/SDS-PAGE was run which showed the presence of the two chimeras at the complex level. Moreover, the other bands of the hrCN gel that corresponded to each fluorescent protein migrated on the SDS-gel at the molecular weight estimated for the protein and the fluorochrome, i.e. 75 and 33 kDa for mCherry-TorC and GFP-TorE, respectively (Fig. 4b). Finally, the bands corresponding to the complex and to each chimera were cut from the hrCN-gel and were analyzed by mass spectroscopy. As expected, since a crude membrane extract was loaded on a gel, many proteins were present but recombinant TorC and TorE were significantly identified in the two replicates with high sequence coverages (Table 1). This analysis indicates that TorE and TorC co-migrate in the gel and that they interact to form a complex. According to the estimated sizes of the complex obtained in the various experiments (434 kDa, 305 kDa) and according to the size of labelled TorE and TorC, we propose that the complex is made up of 4 TorE and 4 TorC (Table 2). A similar experiment was performed with the proteins of the Nap system. NapC and NapE were labelled with GFP and mCherry, respectively, and produced together in *S*. *oneidensis*. The membrane extract of the recombinant strain was submitted to 3–14% hrCN-PAGE. Analysis of the fluorescence of the gel indicated that a complex is formed by the two proteins as visualized by the yellow color of the band. Based on the molecular weight of each labelled protein (47.6 and 32.5 kDa for GFP-NapC and mCherry-NapE, respectively) the complex has a mass of 336 kDa corresponding to a stoichiometry of 4NapE:4NapC. This result corroborates those obtained with TorE and TorC and indicates that both TorE and NapE are involved in the anchoring of their cognate cytochrome into the membrane.

**Table 1:** Protein content identified by mass spectrometry in the 430 kDa band sample.

Order^a^	Accession^b^	Description^c^	Coverage %^d^	PSM^e^	Unique Peptides^f^	Score^g^
1	Q8E8H2	proton-coupled multidrug efflux pump permease component VmeB	29	45	21	137
3	—	mCherry_TagGS_TorC	39	39	19	114
13	—	GFP_tagGS_TorE	25	12	4	31

^a^Order in the identified proteins.

^b^Uniprot accession number.

^c^Uniprot Description.

^d^Protein sequence coverage by the matching peptides.

^e^Peptide spectral matches given by the algorithm corresponding to the total number of identified peptide sequences.

^f^Number of different peptides matching to protein sequence and unique to this protein.

^g^Protein score given by Sequest algorithm.

**Table 2:** Theoretical molecular weights (kDa) of the complex with various stoichiometries of the subunits.

	GFP-TorE + TorC or GFP-TorC + TorE	GFP-TorE + mCherry-TorC
1: 1	77,69	104,67
ΔMW	221,23	329,41
2: 2	155,38	209,33
ΔMW	143,54	224,75
3: 3	233,07	314,00
ΔMW	65,85	120,08
4: 4	310,77	418,67
ΔMW	−11,84	15,41

“ΔWM” stands for the difference between the experimental and the theoretical size of the complex (305 kDa, 434 kDa).

**Figure 5:**
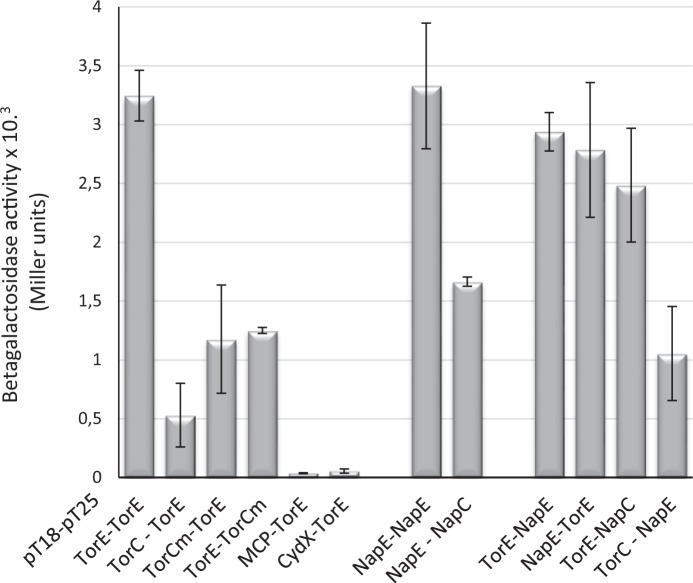
*In vivo* interaction of the proteins using bacterial two-hybrid system. Coding sequences of the studied genes were cloned in bacterial two-hybrid plasmids pT18 and pT25. Recombinants plasmids were introduced in *E. coli* BTH101 in different combinations as indicated on the graph. β-galactosidase activity was assayed on cells grown overnight in the presence of IPTG and was expressed in arbitrary units^62^.

Through a two-hybrid experiment, the dimerization of TorE was confirmed as well as the interaction between TorE and the membrane anchor of TorC (Fig. 5). Moreover, it showed that TorE interacts with NapE to form heterodimers and with NapC. NapE dimerizes and interacts with NapC and with TorC. The specificity of the interactions was ascertained using CydX, as a small protein control, and MCP2117, as a membranous protein unrelated to respiratory systems. In both cases, interaction was seen with neither TorC nor TorE (Fig. 5). Altogether these data confirm that TorE and TorC can be organized as a complex as seen on hrCN gels, and explain why NapE can complement a *torE* mutation and not CydX.

## Discussion

The cellular role of small proteins is still not fully understood. Concerning small proteins related to membranous complexes, they seem to act in the stabilization of the complex although direct experiments are sometimes lacking. However, in various cases like in the Photosystem II of Cyanobacteria, small proteins included in the complex were reported as involved essentially in the stabilization, the assembly, the dimerization or the photoprotection of the complex^43^. In the case of the K^+^ transporter system KdpABC of *E*. *coli*, it was described that the presence of the small protein KdpF is required for the complex activity *in vitro*, can be replaced by an increase of the lipid concentration in the sample and is not necessary for the activity *in vivo*, strongly suggesting that KdpF stabilizes the KdpABC complex in the membrane^44^. CydX is another example of a small protein involved in a membranous complex, CydAB, which is a respiratory system present in many bacteria. According to the bacterium, the role of CydX differs according to the authors from acting on the stability of the complex to a direct role in the quinol binding site of the complex or the coordination of a heme^1,11,12^.

**Figure 6:**
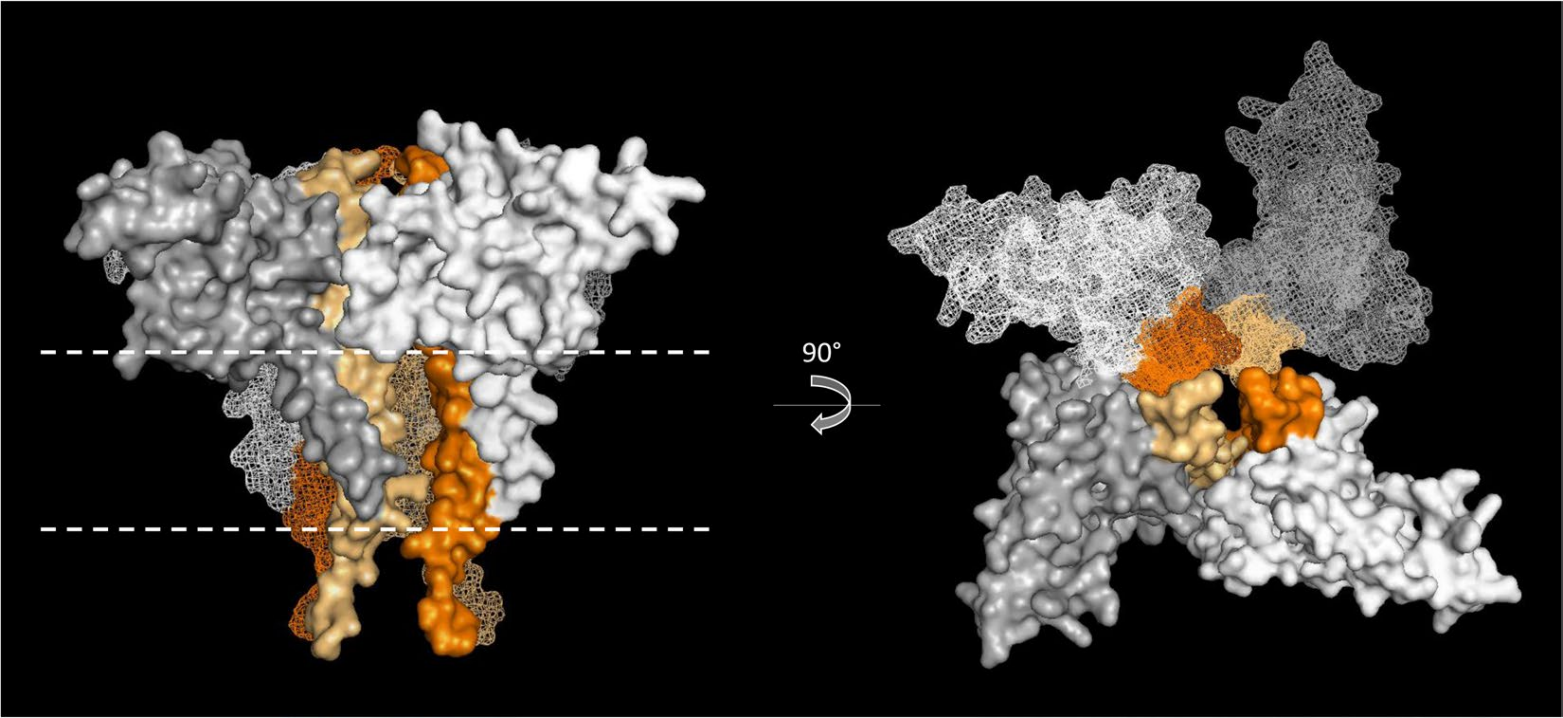
Structure modelling of the TorC – TorE complex. TorC and TorE structures have been modelled with SWISS MODEL^63–67^ based on NrfH an PilN crystallographic structures^68,71^. The presented modeled complex is composed of 4 TorE -TorC heterodimers predicted by Memdock^70^ The TorC proteins are colored in gradient of grey and the TorE in a gradient of orange. Position of the membrane, determined by the PPM server^69^, was represented by dashed white lines.

Here we deciphered the role of TorE, the small protein involved in the Tor system that allows *S*. *oneidensis* growth on TMAO. It was established previously that TorE increases the efficiency of the electron transfer during TMAO reduction^14^. The present study shows that TorE stabilizes the pentahemic *c*-type cytochrome TorC *in vivo* by organizing a complex that contains 4 TorE and 4 TorC (Fig. 4). TorE forms multimers as seen on hrCN gel in the absence of TorC while in the presence of the cytochrome high levels of oligomerization are lost. This indicates that *in vivo* TorE interacts with TorC rather than forming homo-oligomeric clusters in the membrane. In contrast, TorC can only weakly oligomerize according to the results obtained in both hrCN gels. This indicates that TorE is necessary to support TorC-TorC interaction. Since the size of the complex in the membrane corresponds to four monomers of each subunit, there are at least two possibilities for the complex formation. One in which the tetramerization of TorE forms the core of the complex followed by the interaction of each TorE with one monomer of TorC; a second in which the four TorE-TorC dimers are made before being organized in the whole complex. In both cases, the final organization leads to a stronger anchorage of TorC to the membrane (Fig. 6).

The presence of the same highly conserved residues in the hydrophobic stretch of TorE and NapE and their localization on one side of the alpha helix suggest that this region is important probably for an interaction with a partner. This is in agreement with the experiment of complementation of a *torE* mutant with *napE*. The comparison of sequences of the hydrophobic N-terminal part of the various cytochromes TorC indicates that we can distinguish those that interact with TorE from the others by the presence of conserved amino acid residues. These residues are localized in one side of the alpha helix of TorC and in a loop located in its soluble part (Figure S4a and S4b). Using a modelling tool to construct a putative model of the TorE:TorC complex, it appears that the conserved motifs of TorE and TorC could be involved in the binding of the two proteins (Figures S4b and S4c). Using these results, a model of the 4TorE:4TorC complex is proposed (Fig. 6). However, this latter is just a static picture of the complex, one can imagine that the binding of the proteins leads to structural modifications that are not taking into account in Fig. 6. Interestingly, NapC sequence alignment indicates that the same conserved motif is found in all NapC proteins (Figure S4a). Although NapE is able to replace TorE, the growth curves are slightly different. This point suggests that the two proteins TorE and NapE have co-evolved with their partner leading to the sequence variations between them.

Since TorC is organized in two domains, one interacting with TorA and the second feeding the reductase TorA with electrons probably by a flip-flop movement, one can imagine that this stronger anchorage increases the efficiency of the electron transfer and that the TorE-TorC complex can transfer electrons to four TorA enzymes at the same time. Moreover, TorC transfers one electron at a time to TorA, while the reduction of TMAO by the molybdenum cofactor requires two electrons^45^. Another possibility could be that the 4TorE:4TorC complex introduces electrons to two TorA enzymes allowing the simultaneous transfer of two electrons to TorA. We suggest that one or the other of these organizations of the different components of the Tor system improve the electron transfer to TorA. Our conclusion is that the complex is made up of four monomers for each subunit. The hypothesis of the presence of another protein in the TorE:TorC complex was discarded in light of the results obtained. Indeed, as shown in Table 2, none of the various simulated stoichiometries is compatible with an additional component. This supports the hypothesis of a complex containing only a tetramer of two components TorE and TorC.

Recently, it was shown that, in *E*. *coli*, NarGHI complex that allows anaerobic growth on nitrate reduction was localized at the pole of the bacterium^37^. Since the clustering of enzymes involved in a same metabolism is described to increase its efficiency, we wondered if TorE could influence the location of the Tor respiratory system in a peculiar region of the inner membrane. Fluorescence microscopy experiments indicate clearly that the TorEC complex is evenly distributed all along the membrane and that the presence of TorE has no effect on the localization of TorC. Moreover, the presence of the terminal reductase TorA and of the substrate (TMAO) do not modify the distribution of the Tor system, meaning that its activity does not act on the location of the respiratory system contrary to what was observed with the NarGHI respiratory system of *E*. *coli*. Finally, since the NapEC complex and the cytochrome CymA present a similar homogenous distribution in the membrane and their terminal reductase is constitutively produced in anoxia, it seems that at least in *S*. *oneidensis*, the respiratory systems are not clustered in a special region of the membrane in anaerobiosis and that a quite simple complex is sufficient to support an efficient electron transfer.

In conclusion, this study unveils the role of TorE, a small protein present in the Tor respiratory system of various bacteria. TorE by stabilizing the anchoring of the system in the membrane increases the efficiency of TMAO respiration of *S*. *oneidensis*. By extending the study to NapE, we showed that TorE and NapE belong to the same family of small proteins involved in major respiratory systems. They are required to organize the protein complex that increases the anchorage of these systems to the membrane. A rapid adaptation to its environment and an improved energetic reactivity is of crucial importance for the bacterium especially when living in a competitive medium. We thus propose that in respiratory pathways, small proteins of this family are starter that organize the quaternary structuration of the system allowing a positive regulation of the catalytic activity at the complex level.

## Methods

### Synteny analysis

The TorE protein sequence from *S*. *oneidensis* was used as a query for a tBLASTn^46^ analysis in each of the groups of proteobacteria. This analysis allows to find annotated or unannotated *torE* and *napE* genes. The two homologs have been identified according to the presence of *torC* and *napC* genes in the *torE* genomic environment. The *tor* and *nap* operons that do not contain *torE* and *napE*, respectively, were identified using *S. oneidensis* TorC sequence as a query for another tBLASTn analysis. Data from both analyzes were manually compared. Degenerated sequences of *torC* and *napC* genes were also taken into account. The DNA sequences have then been translated using Emboss Transeq online tool^47^.

For construction of the phylogenic tree of proteobacteria, GyrB protein sequence from *S*. *oneidensis* was the query for a tBLASTn analysis of each of the groups of proteobacteria. Only sequences from complete genomes were used. The sequence of the best match was manually taken and translated using the Emboss Transeq online tool^46^. Only one random sequence per genus was kept for construction of the tree and the name of the genus only is indicated. The minimum evolution tree was constructed with the MEGA program^48^ using default parameters. The group of proteobacteria of each species was assigned according to the taxonomy option on the NCBI website^49,50^.

The Sequence Logo has been constructed using the Weblogo online tool^51^ with default parameters using the conserved E residues as first residue.

### Helical projection

The helical projection of residues was carried out using the “Helical Wheel projection” online tool (default parameters) using the sequence of *S*. *oneidensis* TorE protein from residue 31 to 52. The conserved residues were selected according to the Sequence Logo of the different TorE and NapE proteins.

### Bacterial strains and growth conditions

The *E*. *coli* strains C600^52^ and 1047/pRK2013^53^ used for conjugation were routinely grown in Lysogeny Broth (LB) medium at 37 °C.

All *S*. *oneidensis* strains used in this study are derivatives of strain MR1-R^54^ (referred as WT and used instead of MR-1 to allow counter-selection by rifampicin in the conjugation experiments) and were routinely grown at 28 °C in LB medium under agitation. The strains used in this article are listed in Table 3. Deletion of the *torC* gene was done as detailed previously^55^.

**Table 3:** Strains and plasmids used in this study.

Strains	Characteristics	Source
C600	F^-^ tonA21 thi-1 thr-1 leuB6 lacY1 glnV44 rfbC1 fhuA1 λ^−^	Ref.^52^
Bth101	*F′ cya-99 araD139 galE15 galK16 rpsL1* (Str^R^) *hsdR2 mcrA1 mcrB1 relA1*	Ref.^60^
MR1-R	*S*. *oneidensis*, wild type strain, Rif ^R^	Ref.^54^
Δ*torE*	MR1-R strain deleted of *torE* gene.	Ref.^14^
Δ*torC*	MR1-R strain deleted of *tor*C gene.	This study
Δ*torEC*	MR1-R strain deleted of *torEC* genes.	This study
Δ*cymA*	MR1-R strain deleted of *cymA* gene.	This study
**Plasmids**	**Characteristics**	**Source**
pBAD33	Vector containing pBAD promoter with a p15A origin of replication	Ref.^72^
pTorE	Coding sequence of the *S*. *oneidensis* TorE inserted in the pBAD33 vector	Ref.^14^
pNapE	Coding sequence of the *S*. *massilia* NapE inserted in the pBAD33 vector	This study
pCydX	Coding sequence of the *S*. *oneidensis* CydX inserted in the pBAD33 vector	This study
pGFP-TorE	Coding sequence of the sfGFP fused to the *S*. *oneidensis* TorE with GSGGSG spanner and inserted in the pBAD33 vector	This study
pGFP-TorC	Coding sequence of the sfGFP fused to the *S*. *oneidensis* TorC and inserted in the pBAD33 vector	This study
pGFP-TorE; mCherry-TorC	Coding sequence of the sfGFP fused to the *S*. *oneidensis* TorE with GSGGSG spanner and and coding sequence of the mCherry fused to the *S*. *oneidensis* TorC with GSGGSG spanner, inserted in the pBAD33 vector	This study
pGFP-TorC; mCherry-TorE	Coding sequence of the sfGFP fused to the *S*. *oneidensis* TorC with GSGGSG spanner and and coding sequence of the mCherry fused to the *S*. *oneidensis* TorE with GSGGSG spanner, inserted in the pBAD33 vector	This study
pGFP-NapC	Coding sequence of the sfGFP fused to the *S*. *massilia* NapC with GSGGSG spanner and inserted in the pBAD33 vector	This study
pGFP-NapC; NapE	Coding sequence of the sfGFP fused to the *S*. *massilia* NapC with GSGGSG spanner and coding sequence of *S*. *massilia* NapE inserted in the pBAD33 vector	This study
pGFP-NapC; mCherry-NapE	Coding sequence of the sfGFP fused to the *S*. *massilia* NapC with GSGGSG spanner and coding sequence of mCherry fused to *S*. *massilia* NapE with GSGGSG spanner inserted in the pBAD33 vector	This study
pGFP-CymA	Coding sequence of the sfGFP fused to the *S*. *oneidensis* TorE and inserted in the pBAD33 vector	This study
pEC86	*CcmABCDEFGH* genes from *E*. *coli* cloned in the pACYC184 vector	Ref.^73^
pEB355	pUT18C derivative, coding for the T18 domain of the adenylate cyclase of B. pertussis	Ref.^60^
pT18 TorE	Sequence coding for *S*. *oneidensis* TorE cloned in frame at the 3 extremity of the sequence coding for the T18 domain into pEB355	This study
pT18 TorC	Sequence coding for *S*. *oneidensis* TorC cloned in frame at the 3 extremity of the sequence coding for the T18 domain into pEB355	This study
pT18 TorCm	Sequence coding for the 1- amino acids of *S*. *oneidensis* TorC cloned in frame at the 3 extremity of the sequence coding for the T18 domain into pEB355	This study
pT18 MCP	Sequence coding for the SO_2117 MCP from *S*. *oneidensis* cloned in frame at the 3 extremity of the sequence coding for the T18 domain into pEB355	This work
pT18 CydX	Sequence coding for *S*. *oneidensis* CydX cloned in frame at the 3 extremity of the sequence coding for the T18 domain into pEB355	This work
pT18 napE	Sequence coding for *S*. *massilia* NapE cloned in frame at the 3 extremity of the sequence coding for the T18 domain into pEB355	This work
pEB354	pKT25 derivative, coding for the T25 domain of the adenylate cyclase of B. pertussis	Ref.^60^
pT25 TorE	Sequence coding for *S*. *oneidensis* TorE cloned in frame at the 3 extremity of the sequence coding for the T25 domain into pEB354	This work
pT25 torCm	Sequence coding for the 1- amino acids of *S*. *oneidensis* TorC cloned in frame at the 3 extremity of the sequence coding for the T25 domain into pEB354	This work
pT25 NapE	Sequence coding for *S*. *massilia* NapE cloned in frame at the 3 extremity of the sequence coding for the T25 domain into pEB354	This work
pT25 NapC	Sequence coding for *S*. *massilia* NapC cloned in frame at the 3 extremity of the sequence coding for the T25 domain into pEB354	This work

When required, media were solidified by adding 17 g.L^−1^ agar. When needed, chloramphenicol and rifampicin were used at 25 μg.mL^–1^ and 10 μg.mL^–1^, respectively. Plasmid induction was carried out with routinely 0.02% arabinose. When indicated, TMAO (40 mM), fumarate (20 mM) and KNO_3_ (2 mM) were added in the medium as alternative electron acceptors. Except in bacterial two-hybrid experiments, all growths were performed in a medium buffered with MOPS (25 mM) to avoid alkalization by TMA production. Arabinose was added in the growth media for induction of WT/pBAD, Δ*torE*/pBAD, Δ*torE*/pTorE at 0.02% and for induction of plasmids Δ*torE*/pNapE and Δ*torE*/pCydX at 0.2%.

### Plasmid construction

All PCR amplifications were performed with Q5 DNA polymerase (New England Biolabs), from *S*. *oneidensis* MR1-R chromosomal DNA for *torE*, *torC*, *cymA*, *cydX* and *mcp2117* and *S*. *massilia*^15^ chromosomal DNA for *napC*, *napE*. For fluorescence experiments, the primers contain a common spanner sequence containing ggaagtggaggaagtgga at its 5′-ends. Genes coding for mCherry and _sf_GFP were amplified from plasmids with complementary spanner sequence at their 3′ ends. Chimeric sequences were constructed by a second PCR by mixing an equimolar amount of the corresponding PCR products and convergent primers. The gene was cloned between the SalI and the SphI restriction sites of the pBAD33 vector, under the control of the inducible P_ara_ promoter. The resulting plasmids were introduced into strain C600^52^ and controlled by DNA-sequencing before conjugation in *S*. *oneidensis* strains. When two genes were carried by the plasmid, a gene amplification was performed and the gene of interest was cloned between XmaI and XbaI restriction sites. The plasmids used in this article are listed in Table 3.

### Fluorescence microscopy

For fluorescence microscopy, cells were grown anaerobically to mid-exponential phase at 28 °C. Living cells are then shifted in buffer and 2 μl was mounted on microscope slide and coverslip. Images were acquired with a Nikon TiE PFS inverted epifluorescence microscope (100 × oil objective NA 1.45 Phase Contrast) and a Hamamatsu Flash4 sCMOS camera. Images were collected with NIS elements software.

### Solubilization experiments

Cells were grown anaerobically at 28 °C in a medium supplemented with MOPS and TMAO in 1L-bootles until exponential phase. Membranes were prepared as previously described^14^ and washed with 20 mM phosphate buffer pH 7.4, 2% glycerol (Buffer A) in the presence of protease inhibitors and re-ultracentrifuged at 110,000 g for 1 h. Pellets were re-suspended in Buffer A supplemented either with 1.5 M NaBr salt incubated for 30 min or with mild nonionic detergent n-dodecyl-β-D-maltoside (DDM) in the same buffer A with a DDM/protein ratio of 2 (g/g) incubated for 20 min and ultracentrifuged. All steps were done at 4 °C. NaBr supernatant was dialyzed using Slide-A-Lyser Dialysis Cassette 3500 MWCO (Thermo Scientific) against Buffer A supplemented with 0.02% DDM. All chemicals were purchased from SIGMA-Aldrich.

Protein concentrations were determined with Lowry modified assay using bovine serum albumin as standard^56^.

For *c*-type cytochromes detection, unheated membrane and solubilized fractions (10 µg) were submitted to 3–14% SDS-PAGE. Apparent molecular masses were estimated using protein ladder (Lonza ProSieve QuadColor). Proteins were transferred onto nitrocellulose membranes with Pierce Power Blotter (Thermo Scientific) using Mixed Range Molecular Weight program. After a one –hour saturation with PBS 3% BSA, the peroxidase activity of hemes was revealed with the SuperSignal West Pico Kit (Thermo Scientific) on Image Quant Las4000 apparatus (GE Healthcare).

### High resolution Clear Native Electrophoresis (hrCNE) and two dimensional (2D) hrCNE/SDS-PAGE

Conserved frozen DDM-solubilized supernatants were supplemented with the same loading buffer used for Blue Native electrophoresis (BNE)^57^ except for the Coomassie Blue G250 dye which was omitted avoiding the quenching of fluorescent dye protein. 20% Glycerol was added to each sample and 0.001% Ponceau S serves as a marker for the front of the gel. To preserve oligomeric states and avoid protein aggregation during electrophoresis, anionic detergent sodium deoxycholate (DOC) 0.05% was added to the cathode buffer (50 mM Tricine, 7.5 mM imidazole/pH 7, 0.02% DDM). Anode buffer was 25 mM imidazole/HCl pH7^58^. hrCN were carried out using a 3–14% linear polyacrylamide gradient and gels were run with constant current (10 mA) for 3 hours (Mini-Protean II; Bio-Rad). After electrophoresis, protein bands were visualized by fluorescence then by *c* type heme detection and finally with PageBlue (Fermentas) and cut out from the gel for mass spectrometry analysis.

For two-dimensional hrCNE/SDS-PAGE, a hrCN lane was cut out of the gel, washed three times for 10 min with migration buffer (Tris 25 mM, Glycine 192 mM) supplemented with 2% SDS and 0.1% 2-mercaptoethanol then washed in the same buffer without any SDS and 2-mercaptoethanol and applied on the top of 12% SDS-PAGE. After electrophoresis, gels were scanned using the Typhoon FLA9500 laser scanner (GE Healthcare) with excitation at 473 and 535 nm, and filter LPB and LPG (GE Healthcare) for GFP and mCherry, respectively.

### Mass spectrometry

Trypsin digestion and peptide mass fingerprints by MALDI-ToF (Matrix Assisted Laser Desorption Ionisation-Time-of-Flight) were performed as previously described^59^. LC-MSMS analyses (Liquid chromatography coupled to tandem mass spectrometry) (Table 1) were performed on a Q-Exactive plus mass spectrometer (ThermoFisher Scientific, Villebon sur Yvette, France) as previously described^59^.

### Bacterial Two-Hybrid experiment

Plasmids used for two-hybrid experiments are listed in Table 3. Bacterial two-hybrid experiments were performed as described^60,61^ with the following modifications. The PCR fragments were cloned into pEB354 and pEB355 vectors, using the EcoRI and XhoI restriction sites, in frame at the 3′-extremity of the sequences coding for the T18 and T25 domains of the adenylate cyclase from *B*. *pertussis*. For interaction experiments, combination of plasmids coding for the T18 and T25 fusions were transformed into BTH101 strains, and the plates (LB agar, kanamycin (50 μg/ml), and ampicillin (100 μg/ml)) were incubated at 30 °C for 2 days. Ten isolated colonies from each plates were used to inoculate 3 ml of LB containing kanamycin (50 μg/ml), ampicillin (100 μg/ml), and isopropyl β-D-thiogalactopyranoside (0.5 mM). After overnight growth at 30 °C, β-galactosidase activity was measured as described before^62^

### Protein structure modelling, protein docking prediction and model construction

The *S*. *oneidensis* TorC and TorE structures were predicted using the SWISS model tool, the structures used being the NrfH cytochrome from *Desulfovibrio vulgaris Hildenborough* and the transmembrane part of PilN from *Myxococcus xanthus*, respectively^63–68^. The TorC and TorE structures were predicted from residue 23 to residue 184 and from residue 1 to residue 50, respectively. The membrane position was determined in TorC and TorE structure models using the PPM server using default parameters^69^. Membrane docking prediction was determined using the Memdock server, also using default parameters^70^. The model has been constructed using the PyMOL Molecular Graphics system.

## Electronic supplementary material

Supplementary Information
