# Supplementary material for: Small membranous proteins of the TorE/NapE family, crutches for cognate respiratory systems in Proteobacteria
Source: Sci Rep. 2018 Sep 11;8:13576. doi: 10.1038/s41598-018-31851-2 (PMC6134056; doi:10.1038/s41598-018-31851-2)
Supplement: Supplementary file 1 — Supplementary Information [file 41598_2018_31851_MOESM1_ESM.pdf]

Small membranous proteins of the TorE/NapE family, crutches for cognate respiratory systems in Proteobacteria

Olivier N. Lemaire[1], Pascale Infossi[1], Amine Ali Chaouche[1], Leon Espinosa[2], Silke Leimkuehler[3], Marie-Thérèse Giudici-Orticoni[1], Vincent Méjean[1], Chantal Iobbi-Nivol[1*]

Supplemental Figures S1 to S4

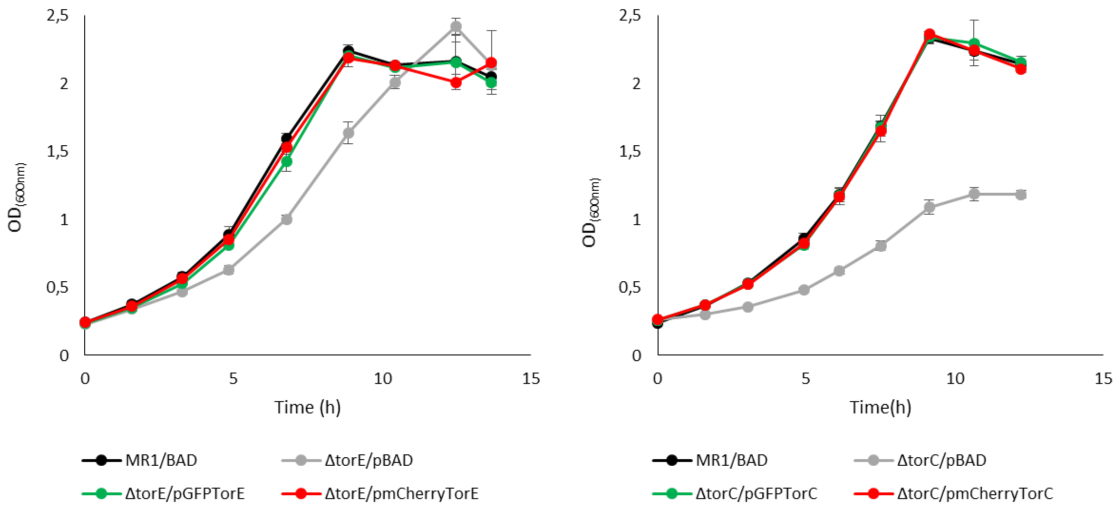

Supplemental figure S1. Complementation experiments. Growth curves of the wild type strain (MR1), Δ*torE* or Δ*torC* mutants harboring either empty pBAD vector or containing sequence coding for N-terminal GFP and mCherry fused proteins. Cells were grown anaerobically in LB medium in the presence of TMAO, arabinose and chloramphenicol. Each point represents the average ± standard error of three independent experiments.

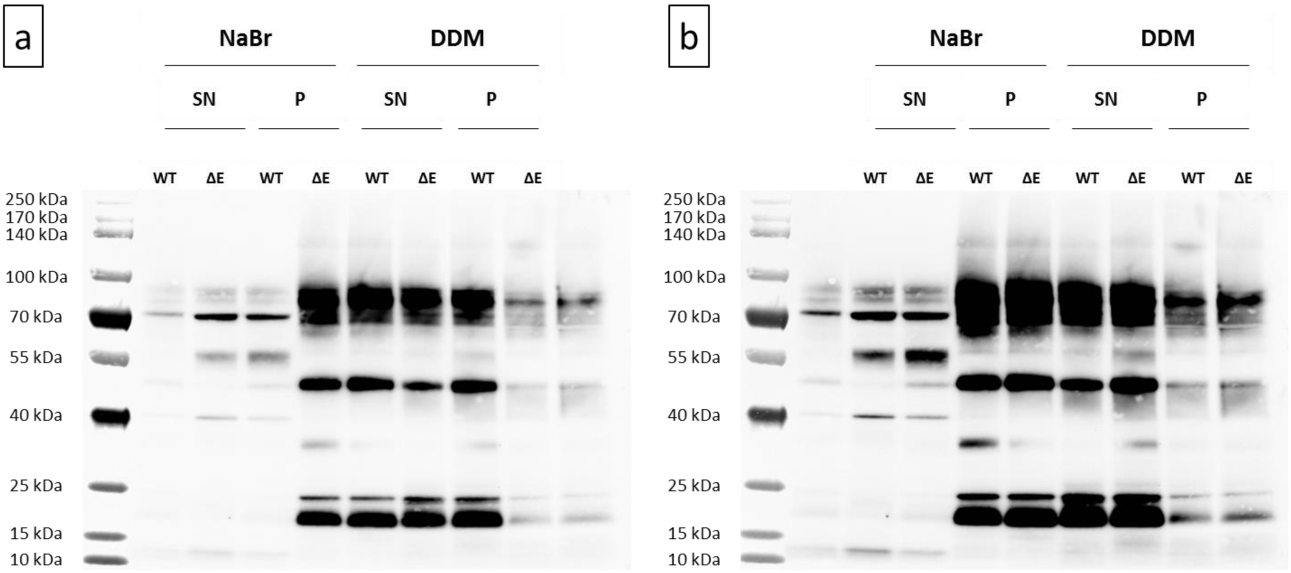

Supplemental figure S2. Entire Blots used for figure 3 construction. a, b. The same gel was submitted to different contrasts in order to visualize the TorC extraction by DDM and NaBr. SN and P stand for the supernatant and pellet fractions after ultracentrifugation, respectively.

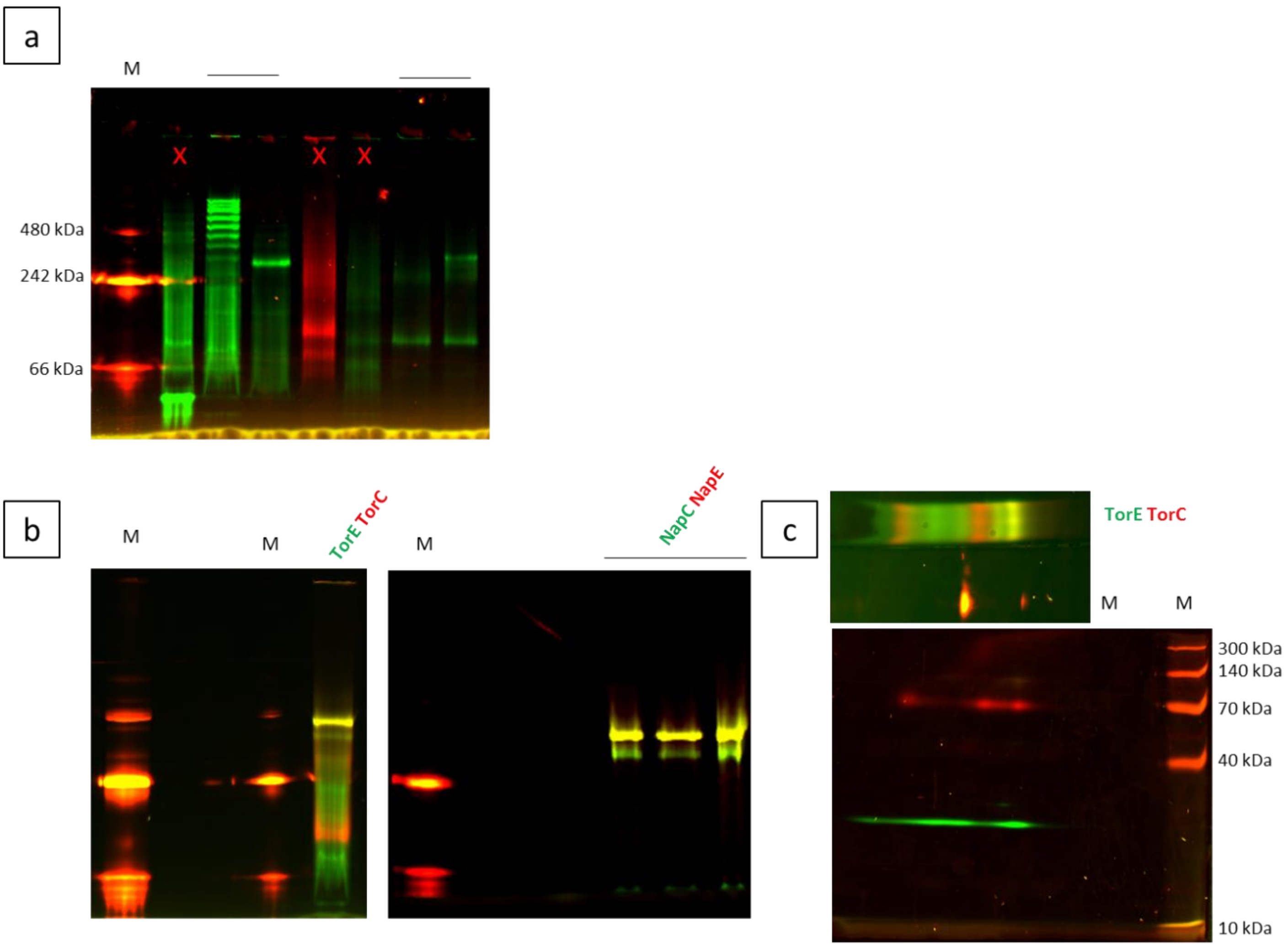

Supplemental figure S3. Entire gels used for figure 4 construction. M is for molecular weight markers. Red x indicate the lanes unmentioned in this article.

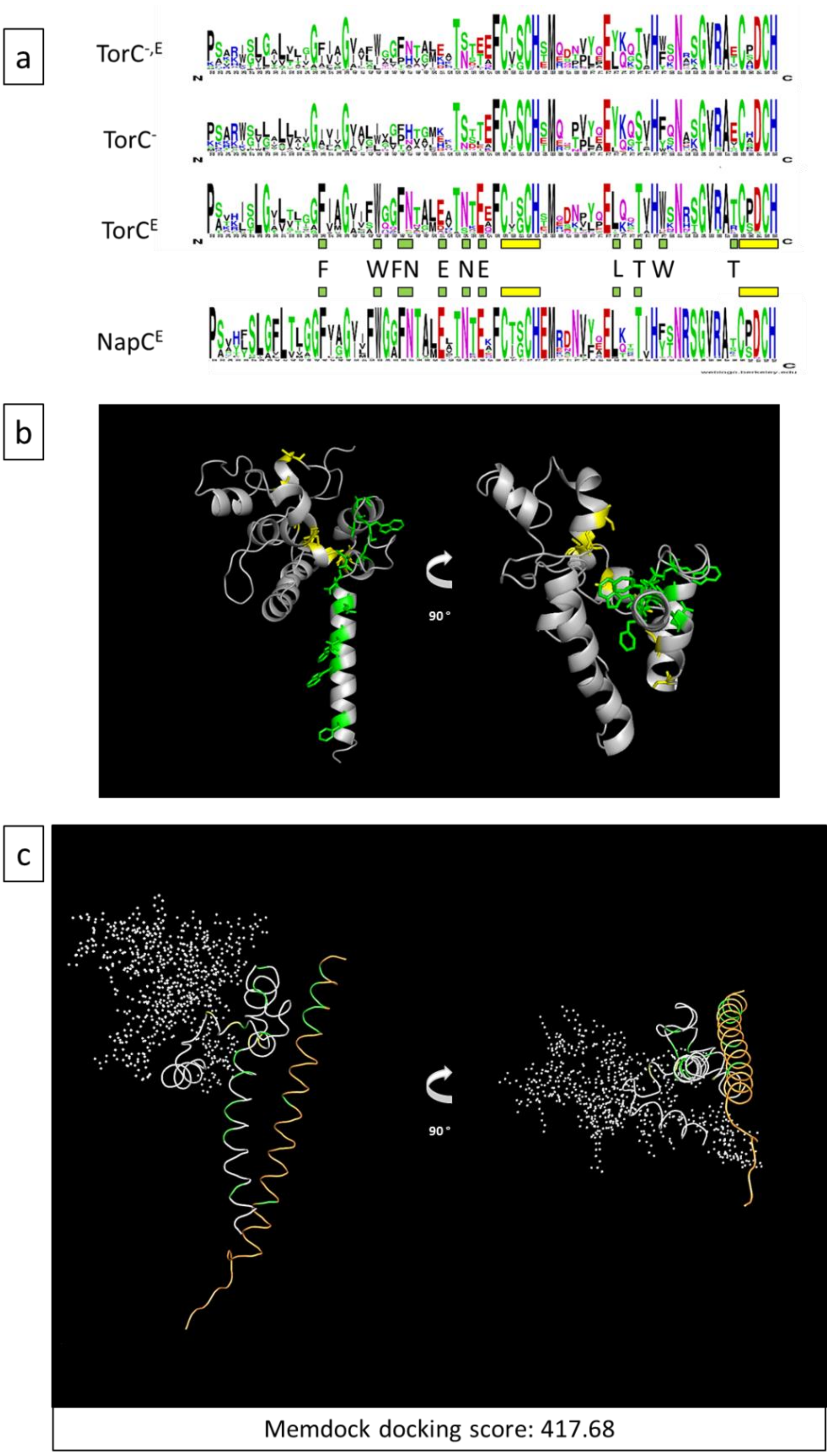
a
TorC-,E
TorC-
TorCE
F WFN E NE L T W T
NapCE
b
90°
c
90°
Memdock docking score: 417.68

Supplemental figure S4. a, b. TorE TorC modelling interaction.

a. Conserved residues in TorC/NapC cytochromes. The sequence logo of TorC was constructed with:
   - all TorC sequences ($TorC^{-,E}$),
   - sequences of TorC independent of TorE, i.e. no TorE-TorC synteny ($TorC^{-}$)
   - sequences of TorC dependent of TorE, i.e. TorE-TorC synteny ($TorC^{E}$)
   - sequences of NapC dependent of NapE, i.e. NapE-NapC synteny ($NapC^{E}$)

   The binding sites of hemes and $TorC^{E}$ conserved residues are indicated by yellow and green blocks, respectively.

b. Position of the $TorC^{E}$ specific residues on *S. oneidensis* TorC structure modeling.

c. Docking prediction of TorE and TorC interaction obtained by the Memdock server. Structure and docking score calculated by Memdock are presented. TorC and TorE are colored in grey and orange, respectively. Cysteines predicted to coordinate the two first hemes are colored in yellow, conserved residues of both protein are colored in green. The chosen docking prediction corresponds to the model with the higher score (first score: 428.71) and involving the specific $TorC^{E}$ conserved residues.
